# Supplementary material for: A yeast phenomic model for the influence of Warburg metabolism on genetic buffering of doxorubicin
Source: Cancer Metab. 2019 Oct 23;7:9. doi: 10.1186/s40170-019-0201-3 (PMC6806529; doi:10.1186/s40170-019-0201-3)
Supplement: Supplementary file 9 — Additional file 9. GO term-specific heatmaps for REMc/GTF-enriched clusters. GO term-specific heatmaps for significant GO process terms were generated as described in methods and Figs. 3 and 4. Any related child terms are presented in subsequent pages of the parent file name. GO terms with more than 100 children, with 2 or fewer genes annotated to the term, or a file size over 300KB are not shown. All heatmaps are generated with the same layout (see Figs. 3 and 4). [file 40170_2019_201_MOESM9_ESM.bz2 › Additional_File9_GOTermHeatmaps/Additional_File9_GOTermHeatmap/regulation_of_transcription_from_RNA_polymerase_I_promoter.pdf]

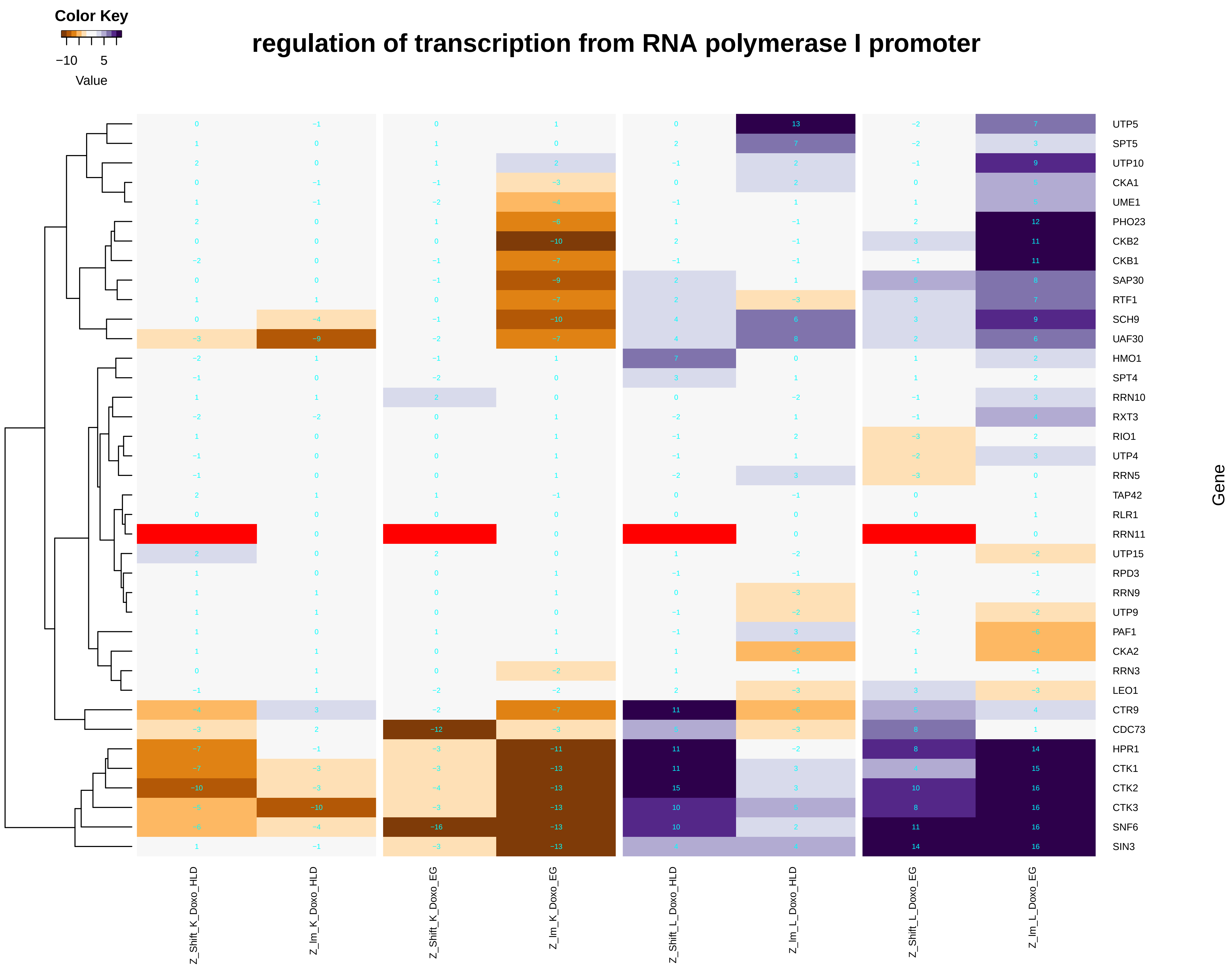
Color Key
−10
5
Value
regulation of transcription from RNA polymerase I promoter
UTP5
SPT5
UTP10
CKA1
UME1
PHO23
CKB2
CKB1
SAP30
RTF1
SCH9
UAF30
HMO1
SPT4
RRN10
RXT3
RIO1
UTP4
RRN5
TAP42
RLR1
RRN11
UTP15
RPD3
RRN9
UTP9
PAF1
CKA2
RRN3
LEO1
CTR9
CDC73
HPR1
CTK1
CTK2
CTK3
SNF6
SIN3
Gene
Z_Shift_K_Doxo_HLD
Z_Im_K_Doxo_HLD
Z_Shift_K_Doxo_EG
Z_Im_K_Doxo_EG
Z_Shift_L_Doxo_HLD
Z_Im_L_Doxo_HLD
Z_Shift_L_Doxo_EG
Z_Im_L_Doxo_EG

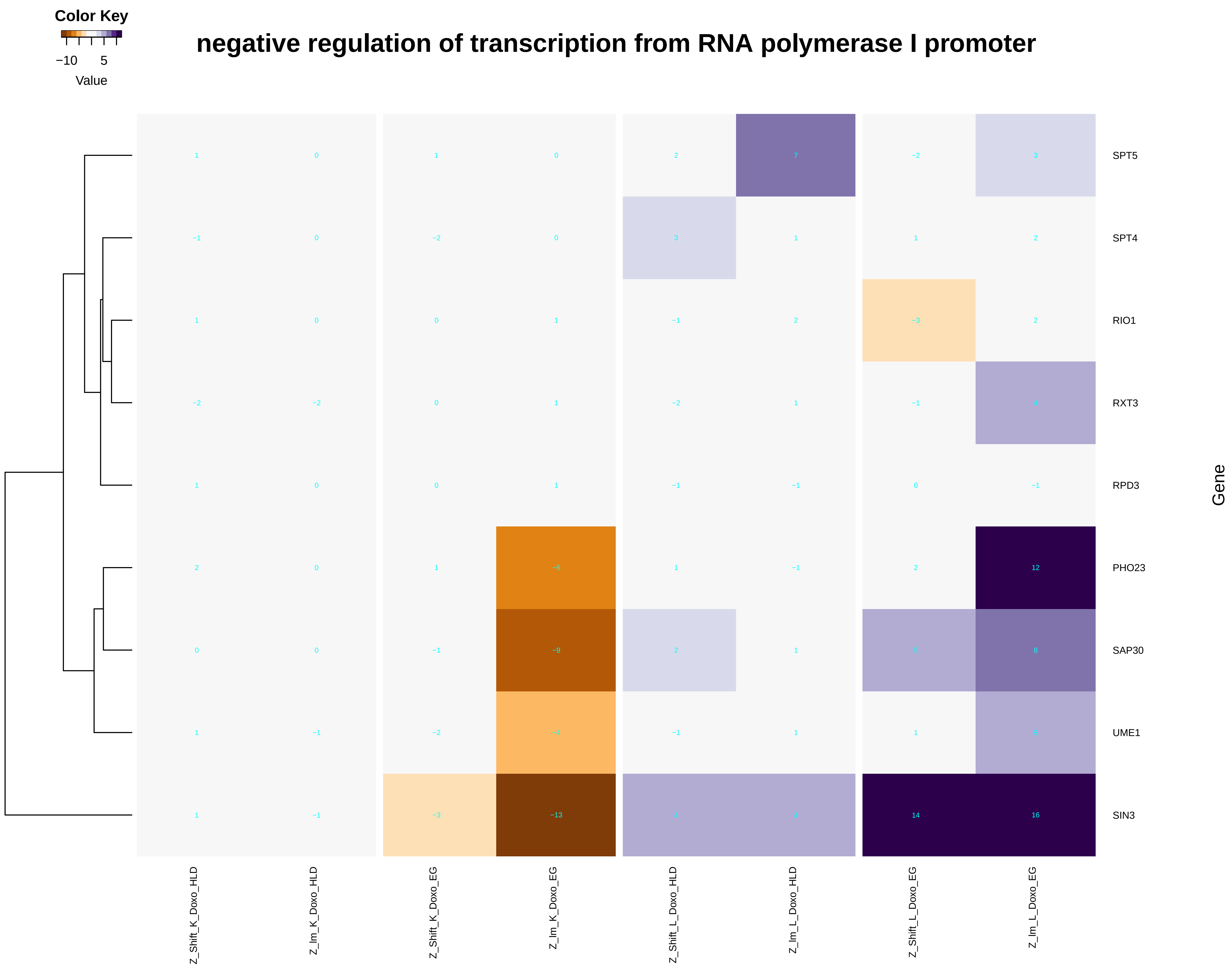

negative regulation of transcription from RNA polymerase I promoter
Color Key
−10
5
Value
SPT5
SPT4
RIO1
RXT3
RPD3
PHO23
SAP30
UME1
SIN3
Gene
Z_Shift_K_Doxo_HLD
Z_lm_K_Doxo_HLD
Z_Shift_K_Doxo_EG
Z_lm_K_Doxo_EG
Z_Shift_L_Doxo_HLD
Z_lm_L_Doxo_HLD
Z_Shift_L_Doxo_EG
Z_lm_L_Doxo_EG

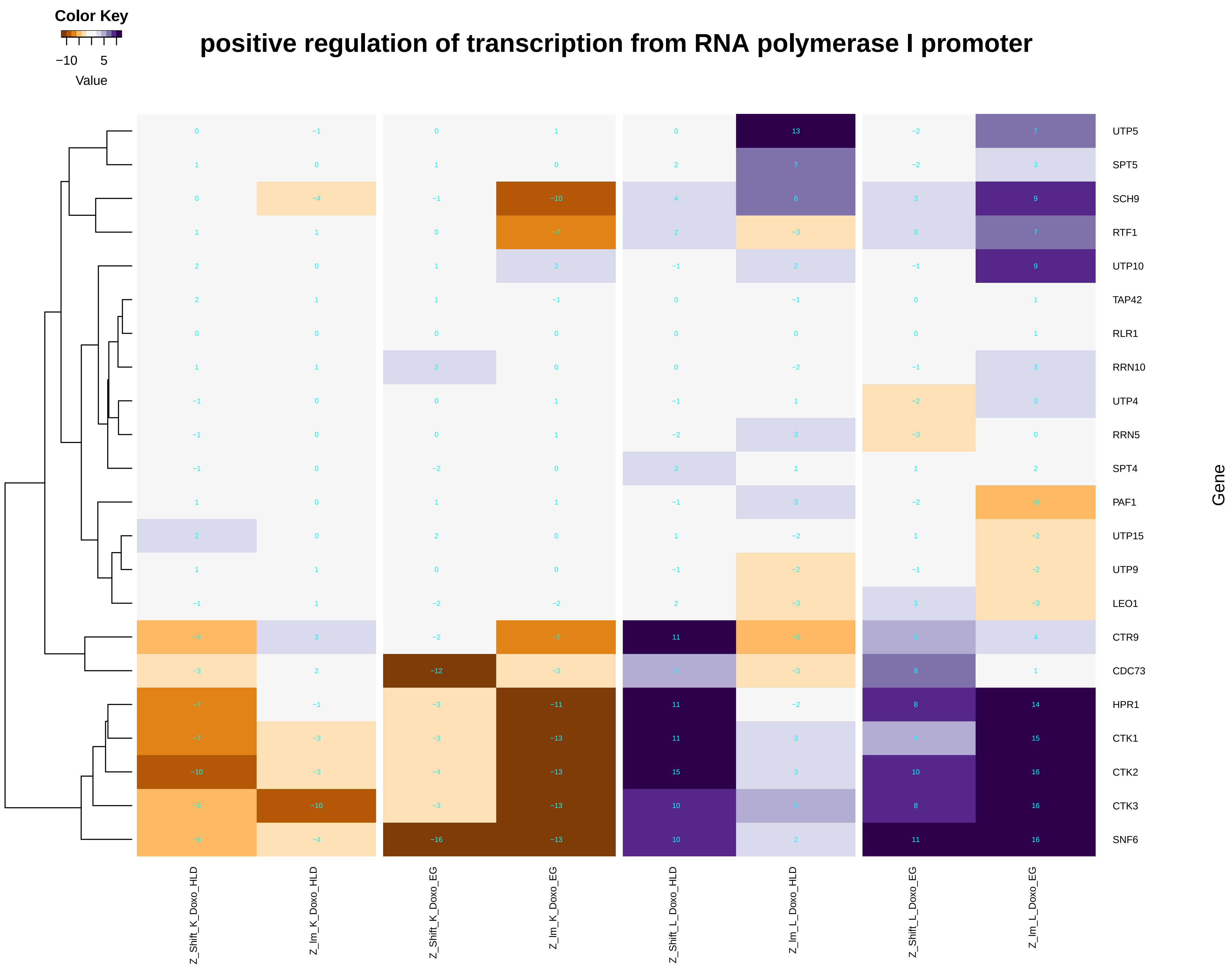
Color Key
−10
5
Value
positive regulation of transcription from RNA polymerase I promoter
UTP5
SPT5
SCH9
RTF1
UTP10
TAP42
RLR1
RRN10
UTP4
RRN5
SPT4
PAF1
UTP15
UTP9
LEO1
CTR9
CDC73
HPR1
CTK1
CTK2
CTK3
SNF6
Gene
Z_Shift_K_Doxo_HLD
Z_lm_K_Doxo_HLD
Z_Shift_K_Doxo_EG
Z_lm_K_Doxo_EG
Z_Shift_L_Doxo_HLD
Z_lm_L_Doxo_HLD
Z_Shift_L_Doxo_EG
Z_lm_L_Doxo_EG

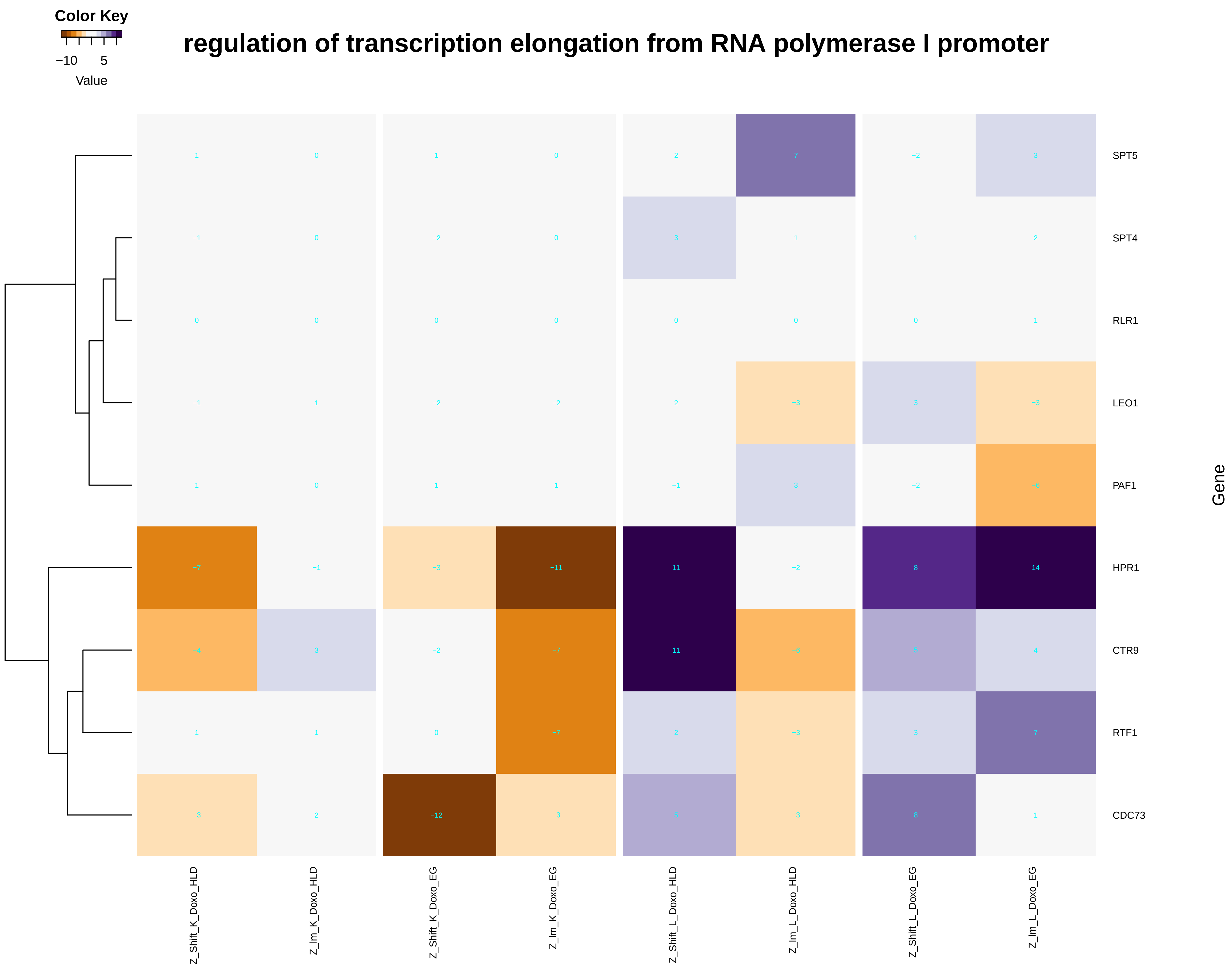
Color Key
−10 5
Value
regulation of transcription elongation from RNA polymerase I promoter
SPT5
SPT4
RLR1
LEO1
PAF1
HPR1
CTR9
RTF1
CDC73
Gene
Z_Shift_K_Doxo_HLD
Z_Im_K_Doxo_HLD
Z_Shift_K_Doxo_EG
Z_Im_K_Doxo_EG
Z_Shift_L_Doxo_HLD
Z_Im_L_Doxo_HLD
Z_Shift_L_Doxo_EG
Z_Im_L_Doxo_EG

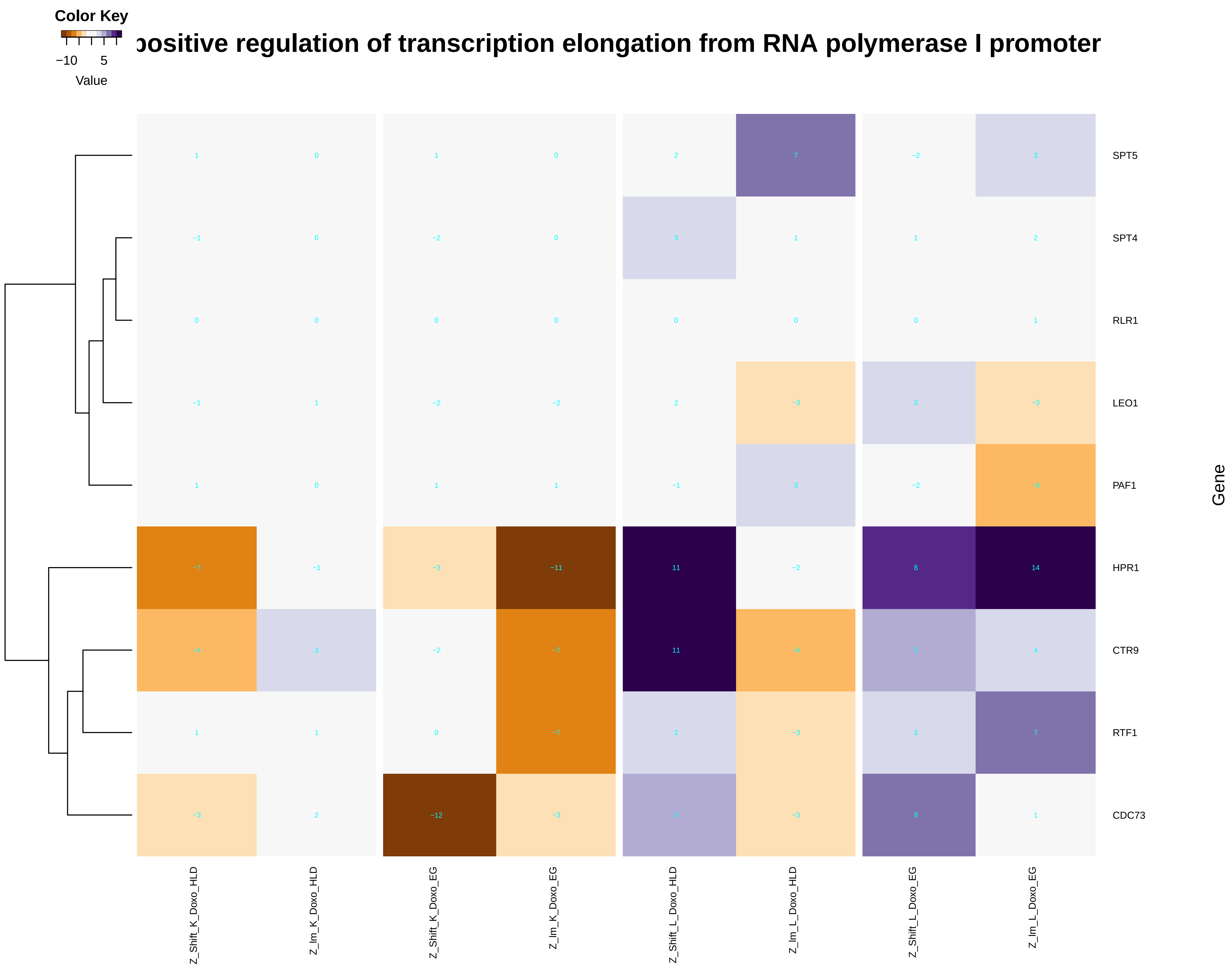
Color Key
−10 5
Value
positive regulation of transcription elongation from RNA polymerase I promoter
SPT5
SPT4
RLR1
LEO1
PAF1
HPR1
CTR9
RTF1
CDC73
Gene
Z_Shift_K_Doxo_HLD
Z_Im_K_Doxo_HLD
Z_Shift_K_Doxo_EG
Z_Im_K_Doxo_EG
Z_Shift_L_Doxo_HLD
Z_Im_L_Doxo_HLD
Z_Shift_L_Doxo_EG
Z_Im_L_Doxo_EG
